# American College of Radiology Thyroid Imaging Reporting and Data System standardises reporting of thyroid ultrasounds

**DOI:** 10.4102/sajr.v24i1.1804

**Published:** 2020-02-06

**Authors:** Mariska Botha, Margaret Kisansa, Wim Greeff

**Affiliations:** 1 Department of Health, Faculty of Radiology, Sefako Makgatho Health Sciences University, Pretoria, South Africa

**Keywords:** thyroid ultrasound, ACR TI-RADS, thyroid nodules, reporting standard, thyroid biopsy, thyroid fine needle aspiration (FNA), improved quality of reports, thyroid sonar, thyroid cancer, thyroid malignancy

## Abstract

**Background:**

Thyroid nodules are common, the majority benign. The small risk of malignancy leads to excessive workup. Thyroid ultrasound is essential for risk stratification and management guidance. Without an organised reporting guideline, reports do not add significant value to referring clinicians. The American College of Radiology Thyroid Imaging Reporting and Data System (ACR TI-RADS) was developed to aid ultrasound reporting, lessen excessive biopsies and diagnose thyroid cancers.

**Objectives:**

To standardise reporting of thyroid ultrasounds by utilising an organised reporting guideline based on ACR TI-RADS.

**Method:**

Thyroid ultrasound reports generated by radiology registrars at an academic hospital were studied in two phases. In Phase 1, the reports were generated as free text, and in Phase 2, using a guideline based on ACR TI-RADS. The percentages of reports that described the maximum size, the five ACR TI-RADS features and a management recommendation were compared.

**Results:**

A total of 130 reports were studied. Significant improvement was observed in the description of all five ACR TI-RADS categories (*p* < 0.0001) from Phase 1 to Phase 2. Of all the reports, 89% included a management recommendation. Reports including an ACR TI-RADS-based recommendation increased from 48% to 75% (*p* < 0.05). Recommendation for biopsy increased from 35.4% to 53.8% (*p* < 0.05).

**Conclusion:**

Introduction of an organised reporting guideline based on ACR TI-RADS, standardised reporting of thyroid ultrasounds by increasing description of thyroid nodule features and ensuring appropriate management recommendations. This, in future, will prevent underdiagnosis of thyroid cancer and unnecessary workup of benign nodules.

## Introduction

Thyroid nodules are common and are detected in up to 67% of asymptomatic patients.^1^ Around 98% of thyroid nodules are benign.^2^ The small malignant potential leads to further imaging, biopsy or diagnostic lobectomy.^3^ This excessive workup leads to over-diagnosis and treatment of subclinical thyroid cancer that would not have altered the outcome.^4^ The 5-year survival rate for thyroid cancer is as high as 98.1%.^5^

There are various classification systems that aid in the management of thyroid nodules, but they are not regularly applied. Risk classification is often based on personal experience or local practice.^6^ There are multiple reporting systems available. One of these guidelines is the Thyroid Imaging Reporting and Data System (TI-RADS), which is based on the American College of Radiology (ACR) Breast Imaging Reporting and Data System (BI-RADS), that has been widely acknowledged in breast imaging.^7^ There are several differences between other systems and the ACR TI-RADS. The ACR TI-RADS does not have subcategories and is user-friendly. It can be implemented as guidelines in voice recognition reporting or computerised clinical decision support systems. Cervical lymph node evaluation is crucial but is not one of the ACR categories. However, the ACR recommends fine needle aspiration (FNA) of suspicious lymph nodes and up to two nodules that meet the criteria for biopsy.^7^

The American Thyroid Association (ATA) system is different in that it uses a pattern-based approach.^8^ The ACR committee opted not to use a pattern-based approach, as a study by Yoon et al. showed that ATA guidelines could not classify all malignant nodules and it was impractical to have patterns for every possible combination of features.^9^ In terms of FNA recommendation, ACR TI-RADS was comparable to other systems for suspicious nodules of 1 cm or more. However, the size cut-off values of the ATA and the Korean Society of Thyroid Radiology for minimally suspicious nodules are lower than that of the ACR.^6,8^ In 2019, the ACR TI-RADS outperformed the other systems in reducing the number of unnecessary biopsies.^10^

The ACR TI-RADS guideline was implemented in two separate steps. In 2015, they published a lexicon with terms to describe thyroid nodules and in 2017, a white paper introduced the TI-RADS risk stratification system and management recommendations based on thyroid nodule size and ultrasound characteristics.^11^ The ACR TI-RADS categorises ultrasound features as benign, minimally suspicious, moderately suspicious or highly suspicious for malignancy. Ultrasound features in a nodule are awarded points and the total score determines the nodule’s ACR TI-RADS level. This varies from a level of one (benign) to a level of five (highly suspicious to be malignant). Owing to the limited availability of sono-elastography, it does not form part of the ACR TI-RADS. The ACR TI-RADS level of a nodule and its maximum diameter determine the recommendation for FNA or ultrasound follow-up. It is designed to balance the cost of biopsy of benign nodules with the benefit of diagnosing cancers (see Figure 1-A1).^11^

Since the publication of the ACR white paper in 2017, two international studies have reviewed ultrasound features of cytologically and histopathologically proven thyroid nodules and validated that the ACR TI-RADS serves as an effective risk stratification tool.^4,12^ Subsequently, a local study conducted in Johannesburg in 2019 that compared thyroid ultrasound features with cytology and histopathology confirmed that using a thyroid ultrasound reporting system improves accuracy.^13^

Without applying an organised reporting guideline, the standard of thyroid ultrasound reporting is poor and does not add significant value to the referring clinician. A study conducted in Johannesburg in 2017 confirmed this by evaluating whether the reports commented on important features that were suspicious of malignancy.^14^ This finding was reiterated by a Canadian study in 2018, which proved that most thyroid ultrasound reports were of poor standard because they provided insufficient information to the referring clinician.^15^ Underreporting of nodule characteristics as recommended by guidelines, leads to delayed diagnosis by cytology and delayed treatment.^16^

Aiming to improve the standard of thyroid ultrasound reporting, a quality improvement study carried out in the United States in 2018 compared the standard of reports prior to and post-introduction of an ACR TI-RADS reporting guideline. It was found that the standard of ultrasound reports and definitive management recommendations improved significantly.^17^ Although most studies validating the implementation of TI-RADS have been conducted by radiologists with substantial experience in ultrasonography, a study carried out in South Korea in 2016 proved that the performance of TI-RADS risk stratification is comparable amongst physicians with different levels of experience.^18^ Although there is an inter-observer variability in radiologists’ interpretation of ultrasound features, it has been shown that using the ACR TI-RADS also improved agreement on biopsy recommendation.^19^

Prior to induction of this study, a trend was observed at Dr George Mukhari Academic Hospital that many thyroid ultrasounds reported by radiology registrars were inconclusive. These reports either provided no management recommendation or relied on referral for thyroid scintigraphy to decide whether biopsy was indicated. For reports to be conclusive and provide valuable feedback to referring clinicians, they need to include description of all necessary characteristics that could predict malignancy and provide management recommendations. The aim of this study was to add to the paucity of data from Africa by standardising reporting of thyroid ultrasounds using the application of an organised reporting guideline. Even though there are a multitude of other systems, this study opted to use the ACR TI-RADS. It served as a reference to the characteristics a reporting guideline should contain, prompting the reporting radiologist on what to look for and report on.

## Research methods and design

### Study design

A cross-sectional design was used in which two groups were analysed in two phases. The first group included the reports generated by radiology registrars prior to application of a standard reporting guideline, and the second group, the reports generated after application thereof. The outcome measured was the standard of thyroid ultrasound reports.

### Setting

Thyroid ultrasound reports were generated by radiology registrars during 2018 and 2019 at the radiology department of Dr George Mukhari Academic Hospital. Each month, there were a different group of registrars working in the ultrasound department, therefore reports were not always generated by the same group of registrars.

### Sampling

In both phases, registrars obtained ultrasound images using linear high-frequency broadband transducers (5 MHz–18 MHz) on Phillips Epiq 5 ultrasound machines. Gray scale static images with or without colour Doppler were saved in the Picture Archiving and Communications System (PACS). Reports were generated based on these images and saved in the PACS.

In Phase 1, thyroid ultrasound reports carried out as free text were studied. Old reports saved on the PACS were retrospectively selected using convenience sampling until a sample size of 65 was achieved. Thereafter, registrars were trained in using the new reporting guideline based on ACR TI-RADS at an academic meeting. Continual reinforcement was conducted by means of educational banners in ultrasound rooms. In Phase 2, thyroid ultrasound reports, post guideline application, were selected prospectively. Reports were collected as new patients were referred to the ultrasound department. With an average of two thyroid ultrasounds requested per week, it took 6 months to reach a sample size of 65 using convenience sampling.

Sample size calculation, carried out by a statistician, was based on the comparison of the percentage of reports that provided management recommendation in Phase 1 and Phase 2. With a sample size of 65 in each phase, a two-sided Fischer’s exact test had a 91% power. Sample size calculation was carried out with nQuary Advanced (Statistical Solutions Ltd, Cork Ireland), Release 8.0.

### Data collection

The thyroid ultrasound reports of both phases were collected. A data collection sheet was completed for each report. Some reports described single nodules and others described multiple. The data collection sheet that was completed for each report, took into consideration whether descriptors where mentioned on all these nodules. Therefore, only one data collection sheet was completed per report, regardless of the number of nodules reported. Selection bias was prevented as the researcher did not select or discard any nodule.

The data collection sheet checked whether the main characteristics that predicted the probability of thyroid malignancy, as outlined by ACR TI-RADS, were described. These characteristics were: echogenicity, composition, margin, shape and echogenic foci. It also checked whether a maximum size was ascribed, a TI-RADS level was assigned and a management recommendation was stated. Management options were any of the following: management not specified, no further management advised, thyroid scintigraphy, follow-up ultrasound or biopsy.

Management recommendations that form part of ACR TI-RADS are either no FNA, follow-up ultrasound or FNA. Scintigraphy is not a part of ACR TI-RADS. Because of incorrect referral for thyroid scintigraphy at Dr George Mukhari Academic Hospital, data on scintigraphy recommendation were also collected.

### Data analysis

The primary outcome measured was a comparison of the percentage of reports in Phase 1 and Phase 2 that described the maximum size, described each of the five ACR TI-RADS features, assigned an ACR TI-RADS level and reported a management recommendation. Significance of the difference in percentages was tested by the Fischer Exact test and results were presented as graphs and tables.

Statistical analysis was performed using SAS (SAS Institute Inc, Cary, NC, USA) Release 9.4, running on Microsoft Windows for personal computer. Statistical tests were two-sided and *p*-values of < 0.05 (5%) were considered significant.

### Ethical consideration

There was no reimbursement for participation of radiologists or patients in this study. Privacy and confidentiality were ensured by anonymising both patients and radiologists involved in each ultrasound report. Consent was obtained from the superintendent of Dr George Mukhari Academic Hospital for collection of electronically stored data from patients’ profiles on the PACS. The research procedure did not entail any intervention directly affecting patients and merely addressed the reporting habits of radiologists. This ensured that there was no risk of harm to the patients involved. For this reason, informed consent was not obtained from the patients. Ethical clearance was granted by the Research Ethics Committee of Sefako Makgatho University on 07 March 2019 (Ethical clearance number: SMUREC/M/41/2019/:PG, REC 210408-003).

## Results

A total of 130 reports were studied. The frequency of reported characteristics of thyroid nodules is shown in Table 1. In Phase 1, most of the ACR TI-RADS characteristics were omitted from reports. Shape was not outlined in 69.2% and echogenic foci in 55.4% of reports. Margin was not mentioned in 47.7% and echogenicity in 43.1% of reports. Composition was not commented upon in 36.9% of reports. Maximum size was the only characteristic that was well reported in both phases. Of the reports in Phase 1, 85.1% described the maximum size.

**TABLE 1:** Thyroid nodule characteristics in Phase 1 and Phase 2.

Characteristic	Frequency (*n* = 65)				*p*
	Phase 1: Pre ACR TI-RADS		Phase 2: Post ACR TI-RADS		
	*n*	%	*n*	%	
**Maximum size**	-	-	-	-	0.3
Described	53	81.5	58	89.2	-
Not described	12	18.5	7	108	-
**Composition**	-	-	-	-	< 0.0001
Described	41	63.1	60	92.3	-
Not described	24	36.9	5	7.7	-
**Echogenicity**	-	-	-	-	< 0.0001
Described	37	56.9	60	92.3	-
Not described	28	43.1	5	7.7	-
**Shape**	-	-	-	-	< 0.0001
Described	20	30.8	54	83.1	-
Not described	45	69.2	11	16.9	-
**Margin**	-	-	-	-	0.0001
Described	34	52.3	55	84.6	-
Not described	31	47.7	10	15.4	-
**Echogenic foci**	-	-	-	-	< 0.0001
Described	29	44.6	60	92.3	-
Not described	36	55.4	5	7.7	-
**ACR TI-RADS level**					< 0.0001
Assigned	23	35.4	47	72.3	-
Not assigned	42	64.6	18	27.7	-
**Management recommendation**	-	-	-	-	0.4
Given	54	83.1	58	89.2	-
Not given	11	16.9	7	10.8	-

ACR TI-RADS, American College of Radiology Thyroid Imaging and Reporting system.

After application of the ACR TI-RADS reporting guideline, there was a statistically significant difference between the two phases in terms of frequency of reports describing echogenicity, composition, margin, shape and echogenic foci (*p* < 0.0001). The most underreported characteristics in Phase 1 were shape and echogenic foci. The description of shape increased from 30.7% to 83.1%. The description of echogenic foci increased from 44.6% to 92.3%. The percentage of reports that assigned an ACR TI-RADS level had a statistically significant increase from 35.4% to 72.3% (*p* < 0.05).

As outlined in Table 1, there was no significant distinction in the number of reports that gave a management recommendation between the two phases (*p* > 0.05). It increased from 83% to 89%. Figures 1 and 2 show management recommendations given by the registrars in Phase 1 and Phase 2 respectively. As demonstrated in Figure 3, the number of reports that gave management recommendations that are part of the ACR TI-RADS (i.e. no biopsy or no further management, follow-up ultrasound and biopsy) increased from 48% to 75% (*p* < 0.002) and other recommendations decreased. Figure 4 shows that the percentage of reports that recommended either biopsy alone or biopsy and scintigraphy increased significantly (*p* < 0.051).

**FIGURE 1:**
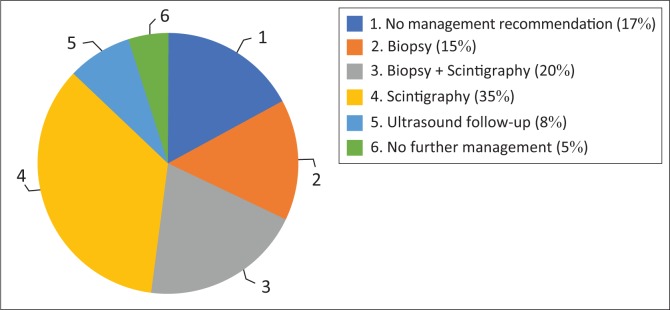
Phase 1 management recommendations.

**FIGURE 2:**
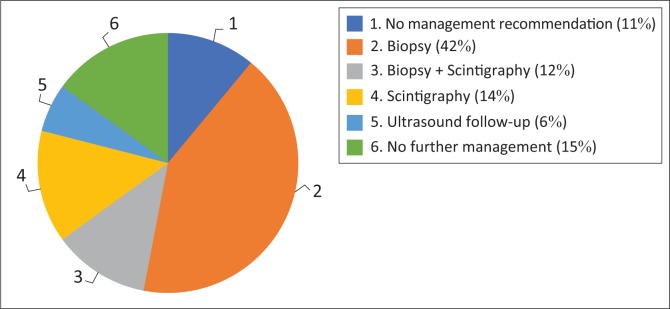
Phase 2 management recommendations.

**FIGURE 3:**
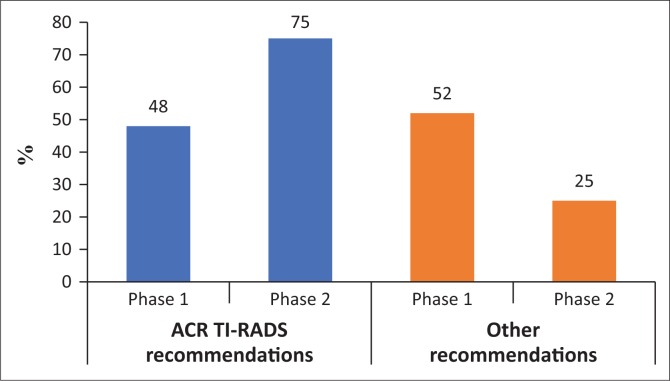
Management recommendations.

**FIGURE 4:**
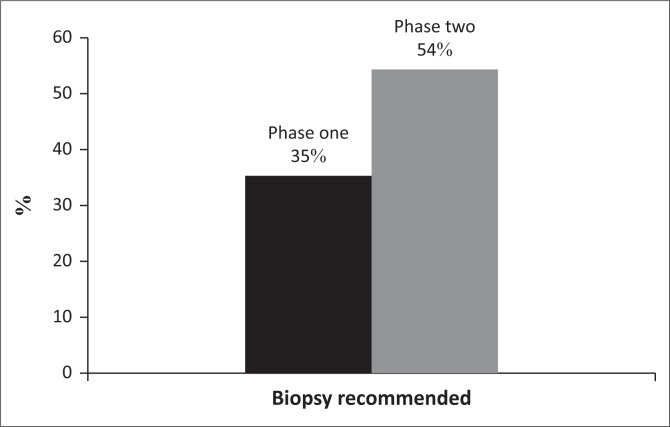
Biopsy recommendation rate.

## Discussion

The reporting of thyroid nodules by radiology registrars at an academic hospital was studied in two phases. The standard of reports prior to using the ACR TI-RADS reporting guideline was compared with the standard of reports with the use thereof. We discovered that application of a guideline significantly expanded the number of reports that included descriptions of thyroid nodule characteristics suspicious of malignancy. This ensured appropriate risk stratification and increased the amount of reports with appropriate recommendations as outlined by the ACR TI-RADS for further management.

Two findings on thyroid ultrasound that are highly specific for malignancy are the presence of microcalcification and direct tumour invasion.^20,21^ These findings are classified under echogenic foci and margin. The description of echogenic foci improved from 44.6% to 92.3%. Reporting on the margin also improved from 52.3% to 84.6%. The underreporting of the most suspicious features of thyroid nodules in Phase 1 led to an underestimation of the risk of malignancy. Improved description of these suspicious features in Phase 2 ensured appropriate risk stratification.

Even though most reports in Phase 1 did include a management recommendation, underestimation of the risk of malignancy meant that these recommendations were inappropriate. Most reports were inconclusive and either provided no management recommendation or recommended thyroid scintigraphy to decide whether biopsy was warranted. With the use of the ACR TI-RADS reporting guideline, more reports had conclusive findings. The incorrect recommendation of scintigraphy decreased from 35% to 14% and the correct management recommendations as outlined by the ACR TI-RADS increased from 48% to 75%.

The European Association of Nuclear Medicine guideline states that thyroid scintigraphy is indicated in patients with a low thyroid stimulating hormone (TSH) level. Hyper-functioning nodules in thyroid scintigraphy have a negative predictive value for malignancy of 96% – 99%. Most thyroid cancers are hypo-functioning. However, up to 90% of hypo-functioning nodules are benign and still need thyroid ultrasound risk stratification based on TI-RADS. Thyroid scintigraphy is further indicated to evaluate thyroid nodules with indeterminate cytology.^22^

In previous studies conducted in developed countries, the implementation of an ACR TI-RADS reporting lexicon decreased the biopsy rate.^17^ In this study, however, the opposite was true. Improved description of suspicious features ensured correct risk stratification and caused an increase in recommendation for biopsy. It is also because the disease profile is different in Africa than in developed countries. Many patients in the African setting present with advanced disease, either as a result of patient-mediated factors (lack of awareness, low level of education or use of alternative medicines) or health service-mediated factors.^23^

The omission of suspicious features was reduced by registrars who were specifically prompted to look for such features using the guideline. Similarly, having management options as part of the guideline aided the registrars to provide appropriate management recommendations. Lack of recommendations or inappropriate recommendations causes unnecessary confusion for referring clinicians. This is also applicable to other imaging modalities. An international study has shown that using a structured guideline that incorporates the Prostate Imaging Reporting and Data System (PI-RADS) lexicon improved the diagnostic standard of prostate MRI.^24^ Similarly, another international study found that utilising a structured Liver Imaging Reporting and Data System (LI-RADS) guideline lead to more consistent reporting of hepatocellular carcinoma features.^25^

There were limitations to this study. Firstly, the participating radiology registrars were aware that their reports were studied. This could have temporarily increased their adherence to using the ACR TI-RADS guideline and introduced an observational bias. Secondly, the reports from only one academic hospital were studied. A multi-centre study is needed for a consensus statement to be issued on the adoption of the ACR-TI-RADS guideline by radiologists and sonologists in South Africa. Lastly, thyroid ultrasound reports were not compared with thyroid scintigraphy reports or biopsy results. Future studies should address this and calculate accuracy, sensitivity and specificity of the ACR TI-RADS.

## Conclusion

Introduction of a reporting guideline based on the ACR TI-RADS, standardised thyroid ultrasound reports. It ensured improved description of thyroid nodule features that are suspicious of malignancy and increased the number of reports with appropriate management recommendations. This would in future prevent the underdiagnosis of thyroid cancer and reduce unnecessary workup of benign nodules.

## Figures and Tables

**FIGURE 1-A1:**
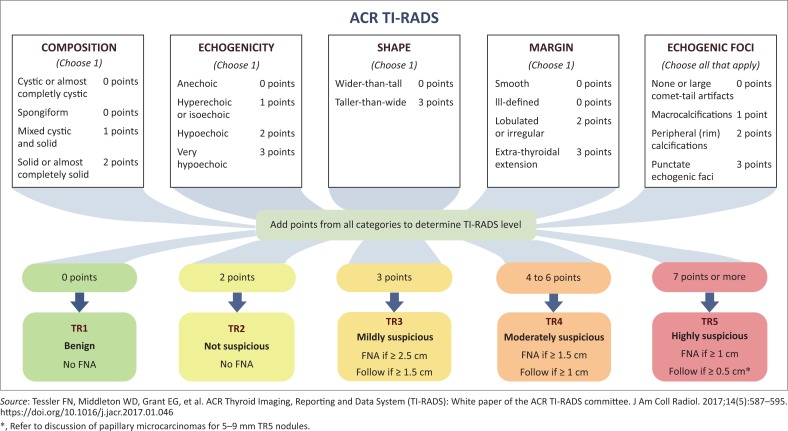
The American College of Radiology Thyroid Imaging Reporting and Data System.^11^
